# An Effective Massive Sensor Network Data Access Scheme Based on Topology Control for the Internet of Things

**DOI:** 10.3390/s16111846

**Published:** 2016-11-03

**Authors:** Meng Yi, Qingkui Chen, Neal N. Xiong

**Affiliations:** University of Shanghai for Science and Technology, Shanghai 200093, China; yimeng56@126.com (M.Y.); xiongnaixue@gmail.com (N.N.X.)

**Keywords:** artificial bee colony algorithm, chaos searching, separation of location and information, virtual data center, wireless sensor network

## Abstract

This paper considers the distributed access and control problem of massive wireless sensor networks’ data access center for the Internet of Things, which is an extension of wireless sensor networks and an element of its topology structure. In the context of the arrival of massive service access requests at a virtual data center, this paper designs a massive sensing data access and control mechanism to improve the access efficiency of service requests and makes full use of the available resources at the data access center for the Internet of things. Firstly, this paper proposes a synergistically distributed buffer access model, which separates the information of resource and location. Secondly, the paper divides the service access requests into multiple virtual groups based on their characteristics and locations using an optimized self-organizing feature map neural network. Furthermore, this paper designs an optimal scheduling algorithm of group migration based on the combination scheme between the artificial bee colony algorithm and chaos searching theory. Finally, the experimental results demonstrate that this mechanism outperforms the existing schemes in terms of enhancing the accessibility of service requests effectively, reducing network delay, and has higher load balancing capacity and higher resource utility rate.

## 1. Introduction

**Figure 1:**
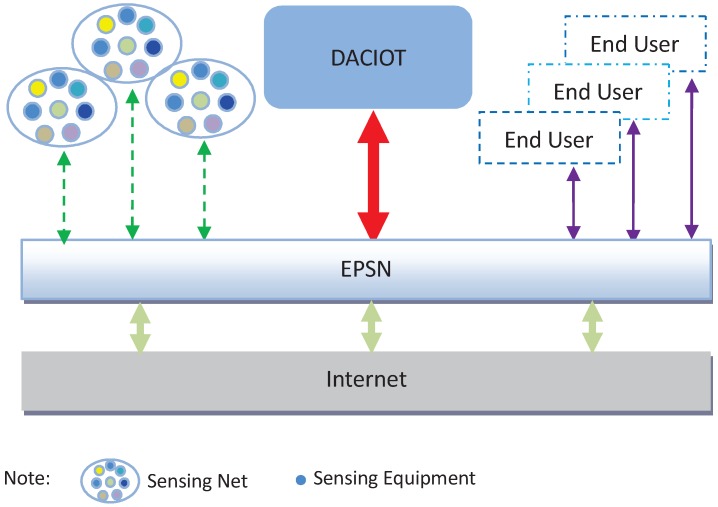
Structure of integration of the IoT application system.

Wireless Sensor Networks (WSNs) are composed of numerous, low-cost micro sensing nodes that are arbitrarily arranged in a monitoring area. It is a multi-hop, self-organizing network system formed by a wireless communication method, with the role of collecting, processing and efficiently transmitting a message from the perceived object within the target range [1,2]. Traditional WSNs have an already rigidified topology structure that is no longer able to deal with the massive and constantly updating application operations [3], henceforth it needs a new-type smart topology structure to solve new challenges brought by data of a huge volume. In various applications of the smart city and modern agriculture, tens of thousands of small WSNs form a large WSN, by which the Internet of Things (IoT) links the relevant information collected on a dynamic basis to the Internet, according to agreement via a large number of intelligent information sensing devices. The collected data are then stored into the Data Access Center for the Internet of Things (DACIOT). After DACIOT’s transformation and control, this information is exchanged and communicated for the intelligent identification, location, tracking, supervision and management of the objects [4] and processes of the application system so that an integrated network is created between man, machine and thing. In the process of integrating such an application system of the IoT, sensors of various types transmit value and non-value types of data. In such a process, a large number of users will send different control requests to the mass sensing devices. In terms of the framework of the IoT application system, DACIOT mainly works at the application level of the upper level of the IoT, shown in Figure 1.

As shown in Figure 1, DACIOT’s key role is to provide network access services for end users (including PCs, mobiles, etc.) and coordinate the massive amount of end users’ requests via transmitting them onto a sensing device. In relation to control demand transmission, as the Internet network has the features of complex structural alternation and fast resource consumption when a massive number of users requests arrive for access, an Effective Path Statistic Network (EPSN) [5] is built and arranged on the Internet, based on the idea of network coverage. EPSN, a logic network comprised of a group of Communication Service Agents (CSA) which has a number of simple functions (data packet sending, receiving, storing and exchange), is a virtual sub-network of the Internet. EPSN explores the effective “empty” path on the Internet based on statistical theory, and measures and calculates the statistics of the measurement parameters and information on the logic chain among CSAs, and builds real-time Effective Paths (EP) based on the measured and statistical data. In this sense, the network that receives the requests for access by the end users is converted from a parameter-uncontrollable Internet network to a transparent and controllable EPSN network. However, the new-type DACIOT is mainly supported by virtual technology [6,7], which is critical to realize cloud computing networks by intensively concentrating and integrating the limited physical resources and providing services of networking, computing and storage in a unified manner. The virtual resources of computing and storing will finally need to provide access to users via the network. In the realm of network, virtualization technology enables a higher utility of the network [8] and makes the network flexibly extendable and manageable. A virtual network allows users of different needs to visit the same physical network. Logically, however, these users are strictly isolated to ensure visiting security [9]. Network virtualization will fulfill the target of improving DACIOT’s [10] operational efficiency, flexibilizing operation arrangement, reducing energy-consumption and releasing rack space.

On the aspect of new-type virtual data center, with virtual integration between physical servers, an increasing number of users choose to rent computing resource in the form of a service from a data center to complete their tasks [11,12], while the users’ huge volume of load of different types can be operated on the servers in different time periods [13]. Largely, these different types of loads can be divided into two types: frequently interactive and non-interactive. Often, the former will initiate emergent operation, e.g., network-based video service with numerous users at night but much less in the daytime; and the latter makes it unnecessary for users to participate further after subscribing a task, e.g., a high-performance computing service. In different periods, these loads will have remarkably different needs in terms of resources [14]. Nevertheless, to ensure that the loads always respond to request for processing, a peak value of needs is always applied to statically allocate enough resources for the loads [15]. Therefore, the majority of server resources are not fully used at the data center, leaving huge hardware, space, energy consumption and management cost wasted. Further, it is very easy to cause imbalance between service access requests, causing part of the server nodes to be offline due to being over loaded [16]. Hence, how to arrange service access requests rationally and efficiently on each of the servers is one of the key challenges to be solved by the data center.

With the aforementioned background, the efforts which have been made for resource optimization at the virtual data center presently are mainly focused on CPU, memory and the network interface. The CPU and memory-based scheduling algorithms focus on reducing as much as possible the fragments in CPU and memory on physical servers on the one hand to be able to contain more virtual machines and enhance the integral utility rate of data center servers [17,18]; on the other hand, it focuses on reserving resources to accommodate the load variation on the virtual machines [19]. These issues of resource allocation can be transformed into typical resource optimization, which can be solved with the swarm intelligent algorithm, heuristic algorithm and greedy algorithm [20]. Network communication is also one of the major factors impacting virtual machine performance [21]. There are currently a large amount of physical servers at a data center, with a comparatively complicated network topology structure. For this reason, the distribution of different structures will severely impact the network communication capacity between physical machines, and further lead to a great gap in the network delay between the virtual machines operating on different physical machines. When this happens, network perception strategy is generally used to solve virtual machine real-time migration and rearrange.

The studies described above are mainly focused on how the virtual data center responds to users’ service access requests through rationally and efficiently arranging virtual machines when a massive amount of user’ requests arrive. However, such studies depend on periodical resource rescheduling by the data center in system operation, which largely impact the stability of the system operations, leading to a sharp rise in the data communication volume, the consumption of a large bandwidth and imposes a heavy burden of network communication upon the DACIOT. Also, when facing a massive amount of user’ service access requests, partial virtual machines can provide very limited resources with their CPU, memory, storage and bandwidth and can no longer consume additional resources to provide services for other virtual machines. Therefore, an urgent task at present is how to allow a huge volume of user’ service requests to be efficiently accessed in the virtualization context, and utilize the resources available at the virtual data center with high efficiency. For this purpose, this paper undertakes deep research on the Distributed Access Problem for Virtual Data Centers (DAPVDC) in relation to massive service requests at the data center. First, the group characteristics and geographical locations of the users are analyzed, based on which an Optimized Dynamic Self-organizing Feature Map Neural Network (ODSOFM) is proposed. This algorithm divides service access requests from different geographical locations into groups logically. Second, by integrally considering the hardware resource like CPU and memory as well as network communication capacity, a Group Migration Artificial Bee Colony Scheduling algorithm (GMBCOS) is proposed from the perspective of service request migration. The approach provides access for various virtual machines after highly-efficient scheduling at the data center using rules. Based on the load of the virtual machines and corresponding service constraints, the information-related logical groups are smoothly migrated from heavy-loaded virtual servers to less-loaded ones so that the data center resources are used efficiently.

The rest of this paper is organized as follows: in Section 2, we overview the related work. Section 3 describes the system model and definitions; Section 4 first puts forward a kind of synergistically distributed buffering access model separating the information on resources and locations, then provides an Optimized Dynamic Self-organizing Feature Maps (ODSOFM) for virtual grouping, and further introduces a Group Migration Bee Colony Optimization Scheduling algorithm (GMBCOS); Section 5 describes the experimental environment, processes and analyses; and Section 6 summarizes the work and describes the anticipated directions for future work in this area.

## 2. Related Work

Regarding traditional massive data access, Apache Kafka (http://kafka.apache.org) represents a quite high throughput with extendibility as a kind of distributed message publishing and subscribing system [22]. It is able to process tens of millions of summary data, both reading and writing requests, per second, and is widely applied to application scenarios demanding high performance, such as massive message queue, log processing and stream processing [23]. The research in [24] demonstrates the diversity in methods and techniques for processing streaming big data in datacentre clouds. The work in [25] designs and implements a hierarchical distributed message queue to guarantee the order of the messages and the exactly once delivery when delivering them to multiple users in parallel. The work in [26] introduces a scalable and mobility-aware hybrid protocol named Boarder Node Medium Access Control (BN-MAC) for Wireless Sensor Networks (WSNs), which leverages the characteristics of scheduled and contention-based MAC protocols. In the research area of virtual data center access, the work in [27] proposes a method that evaluates the impact of virtualization on the network performance of the data center of Amazon EC2 (Elastic Cloud Computing). This method measures the performance impact on processor sharing, data packet delay, TCP/UDP (Transfer Control Protocol / User Datagram Protocol) throughput and packet lost when accessing the virtual machine of the EC2 data center. The research in [28] builds a heterogeneous resource access structure by strongly integrating the multi-layer resources of the heterogeneous data center from the perspective of a software-defined network, and designs a synergistic communication mechanism for the users’ access process, which has some advantages in terms of blocking rate and resource occupation rate. The work in [29] analyzes the characteristics of the virtual data center and proposes an abstracted data center virtualization structure, based on which a flexible and bandwidth-guaranteed allocation algorithm is designed, which further enhances the applicability of the network when being accessed. The research in [30], from the perspective of the service reliability of the virtual data center, proposes a self-adaptive backup strategy based on a genetic algorithm. This strategy can effectively reduce backup time and enhance users’ access request stability via combining different strategies. The work in [31] presents a model that can express the computing, memory, storage and communication resources of the cloud computing data center, which can enhance data center access performance and reliability, from the perspective of cloud infrastructure management and optimization. The work in [32] proposes a powerful meta-heuristic, greedy randomized adaptive search procedure, augmented by path re-linking. By re-optimizing the communication paths between virtual machines and big data sources, this approach re-balances the overall communication and runtime resource utilization on the cloud infrastructure. In order to minimize data access latencies and bandwidth costs, the research in [33] presents optimal algorithms for determining the Virtual Machines (VM) locations satisfying various constraints. The work in [34] proposes a mixed virtual machine sharing and storing system with the consideration of privacy protection when accessing the massive cloud computing data center, and for the purpose of improving I/O (Input/Output) delay and platform throughput. The work in [35] analyzes password system visiting control based on attribute encryption in the context of cloud computing. Moreover, it discusses multi-tenancy technology as well as the virtual visiting control technological framework. The work in [36] designs an agile data center based on virtualization technology of service and storage, and further proposes a load-balancing algorithm for cross resource layers (server, exchanger and storage) with the inspiration of the multi-dimensional knapsack problem. Combining the resources of the data center including CPU and memory, the work in [37] proposes a task scheduling algorithm with bandwidth perception with a consideration of task scheduling of cloud computing. The work in [38] presents two scheduling algorithms for precedence-constrained parallel Virtual Machines (VM) in a virtualized data center where each VM represents a sub-service of the Internet-scale service. The work in [39] designs a rolling horizon scheduling structure for the virtual data center, based on which an energy-perceivable real-time task scheduling algorithm is developed. The work in [40] formalizes the paradigm of big data stream mobile computing, discusses its most significant application opportunities, and outlines the major challenges in performing real-time energy-efficient management of the distributed resources available at both mobile devices and Internet-connected data centers. The literature in [41] proposes a resource management method which is based on feedback control theory and dynamically adjusting the virtual machine resource allocation at the virtual data center, and finally realizes applicable service-oriented resource management via arbitrating various allocation requests for application resources. The work in [42] presents a highly efficient and controllable virtual machine online migration scheduling algorithm to tackle the visiting block problem caused by unreasonable application for core network and unbalanced workload at the virtual data center. The proposed WRR (Weighted Round-Robin) algorithm in [43] is a weighted scheduling algorithm based on polling, and it distributes the service requests between servers in the form of polling. In the aforementioned studies, Kafka is an access mechanism organizing messages in the form of “topics“. However, upon a massive number of service requests at the data center, the problem with the DAPVDC is that it has the feature of a higher complexity of user request types and a strict request response sequence in this scenario. When dealing with the DAPVDC, Kafka consumes a large cost to frequently create the topic and maintain the original order of the service request, limited by its own access schema features though it is widely applied in streaming data processing. WRR in [43] handles a large number of service requests by the means of polling. Although it satisfies the processing fairness of the server requests, it does not make full use of the access ability of new servers, which affects the capacity of the access cluster’s load balancing when processing requests for a longer time. The research in [23,24] mainly considers the real-time processing; the work in [25,26] centres on the reliability of message transmission; the work in [27,28] mainly considers the network resource quality’s impact on access to the virtual data center; the proposed methods in [29,30,31] concentrate on network availability and data center stability; the research in [32,33] mainly centres on the optimization of the network resources cost between virtual machines; the methods in [34,35] focus on privacy protection and visiting safety; the work in [36,37,38,39] mainly consider data center access from the aspect of task optimization and load scheduling; and the work in [40,41,42] solves access problems from the perspective of virtual data center resource reallocation and virtual machine migration.

## 3. System Model and Definitions

In this study, our objective is to solve the problem of DAPVDC in the scenario of the IoT’s large-scale request access.

**Figure 2:**
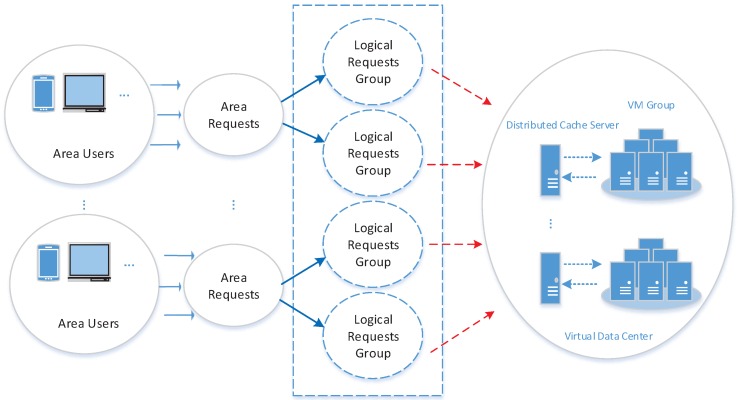
Synergistically distributed buffering access model.

Figure 2 illustrates a synergistically distributed buffering access model with resource and location being separated at the virtual data center. Users in different geographical locations (within the same residential area, for instance) send service requests of different sizes, which can be divided into a logical request group. These requests sent from the same area are deployed into one group, and if the number of requests from this group exceeds a threshold, then the group will be subdivided into two logical request groups based on a related property. Users’ requests from different locations are gathered into the same one logical request group based on the deviation degree between them and the designated threshold and their properties, and so forth, till there are quite a number of logical request groups. The distributed buffering system stores the mapping relation between the logical request group and the virtual machine as well as maintains the correlation before and after the logical request group migration. These logical groups, firstly, search for the mapping relation between the logical request group within the location and the virtual server based on the distributed buffering system, further, searches for the service resources for the logical request groups. If the virtual server in the mapping is overloaded, the other requests in the logical request group will be automatically migrated to other less loaded servers in the virtual server group, and meanwhile, the mapping relation of the distributed buffering system is renewed so that the sequential logical request group can acquire service resources with high efficiency. The detailed definitions of the system model are given in the following paragraphs.

Given the virtual data center network at the time of *t* as N(G,V,P,L), where *G* refers to the collection of virtual logical groups of users’ request, and $G=(g_{1},g_{2},\ldots ,g_{m})$; *V* refers to the collection of virtual machine groups of the data center, and $V=(v_{1},v_{2},\ldots ,v_{n})$; *P* refers to the collection of physical servers of the data center, and $P=(p_{1},p_{2},\ldots ,p_{s})$; and *L* refers to the collection of network links of the data center, and $L=(l_{1},l_{2},\ldots ,l_{d})$. Presuming the physical server $p_{a}$ ($p_{a}\in P$) works normally, then S(a)=1, otherwise, S(a)=0. Assuming that the logical group of users’ request $g_{i}$ requests to access virtual machine group $\upsilon_{u}$, which has $\kappa_{u}$ virtual machines and each machine $v_{u}^{j}$ ($v_{u}^{j}\in V$) has specific requirements on the CPU, memory and storage space, which is referred to as $R_{u}^{j}$; the gross resources of the physical server $P_{a}$ is referred to as $Q_{a}$; the gross communication volume that the virtual machines $v_{u}^{j}$ and $v_{u}^{\kappa }$ pass the collection of link set *L* at the time of Δt is referred to as $c_{u}^{jk}$, and $c_{u}^{jj}=0$; $C_{u}$ refers to the communication matrix of the virtual machine group $\upsilon_{u}$ and it is composed of $c_{u}^{jk}$. The network communication resource that is consumed by accessing number *i* request logical group $g_{i}$ to the number *j* virtual machine $v_{u}^{j}$ is $\psi_{i}^{uj}$, while CPU and memory consumed is $\delta_{i}^{uj}$. Assuming the logical group of users’ request $g_{i}$ is successfully accessed to the virtual machine $v_{u}^{j}$, then $\tau_{u}^{j}\left(i\right)=1$, otherwise, $\tau_{u}^{j}\left(i\right)=0$. In such a case, viable request access means the logical group of users’ request $g_{i}$ can be expressed as:

(1) $$\Phi_{i}=\left[\tau_{u}^{1}\left(i\right),\tau_{u}^{2}\left(i\right),\cdots ,\tau_{u}^{j}\left(i\right),\cdots ,\tau_{u}^{\kappa_{u}}\left(i\right)\right]$$

$$\;s.t.\left\{\begin{array}{c} \tau_{u}^{j}\left(i\right)\in \left\{0,1\right\},j=\left\{1,2,\cdots ,\kappa_{u}\right\}\\ \sum_{j=1}^{\kappa_{u}}\tau_{u}^{j}\left(i\right)=1,\forall i,u\\ \sum_{j=1}^{\kappa_{u}}\tau_{u}^{j}\left(i\right)\cdot R_{u}^{j}\leq \sum_{a=1}^{s}S\left(a\right)\cdot Q_{a},\forall a,u\\ i=1,2,\cdots ,m\\ u=1,2,\cdots ,n \end{array}\right.$$

where $\sum_{j=1}^{\kappa_{u}}\tau_{u}^{j}\left(i\right)\cdot R_{u}^{j}\leq \sum_{a=1}^{s}S\left(a\right)\cdot Q_{a}$ means the available resource quantity of virtual machines in the DACIOT is less than that of physical machines. To make it easier to follow the viable request access means proposed above, an example is taken. $\Phi_{1}=\left[1,0,0,\cdots ,0\right]$ represents the first logical request group $g_{1}$ is successfully accessed to the first virtual machine. Then, the viable request access means of all the logical request groups can be expressed as $\Phi =\{\Phi_{1},\Phi_{2},\cdots ,\Phi_{m}\}$.

It is assumed that Cost(i) means the resource cost consumed when the logical group of users’ request $g_{i}$ requests access to the virtual data center, and

(2) $$Cost\left(i\right)=\sum_{u=1}^{n}\sum_{j=1}^{\kappa_{u}}\left(\tau_{u}^{j}\left(i\right)\cdot \left(\psi_{i}^{uj}+\delta_{i}^{uj}\right)\right)$$

As request groups will migrate between virtual machines, if the load on the virtual machines is too large when a massive number of users request a specific virtual machine cluster, a communication constraint on migration is needed and the network communication resources occupied by migration must not exceed that when requests are accessed, i.e., $\sum_{k=1}^{\kappa_{u}}c_{u}^{jk}<\psi_{i}^{uj}$. Moreover, $\varphi_{j}^{k}\left(i\right)=1$ means that the logical group of users’ request migrates from the virtual machine $\upsilon_{u}^{j}$ to $\upsilon_{u}^{k}$, otherwise $\varphi_{j}^{k}\left(i\right)=0$, and j≠k, $\sum_{k=1}^{\kappa_{u}}\varphi_{j}^{k}\left(i\right)=1$. As the main cost which is incurred when requests migrate depends on the size and communication consumption of an individual request group, the function for average cost of migration is:

(3) $$Mig\left(i\right)=\frac{\sum_{k=1}^{\kappa_{u}}\left(\varphi_{j}^{k}\left(i\right)\cdot \left(1+\lambda \right)\cdot S\left(i\right)\cdot c_{u}^{jk}\right)}{\Delta t}$$

In the equation above, S(i) refers to the size measurement of the logical group of users’ request; *λ* is an adjusting factor which balances the impact of other resources such as CPU and memory on request migration, and λ∈(0,1). Therefore, the problem of DAPVDC can be described in the form of looking for a group of access array $\Phi_{1},\Phi_{2},\cdots ,\Phi_{m}$ within the time of Δt, and a corresponding constraint will be met to minimize the overall resource consumption(minimum is defined as optimal), i.e.,

(4) $$minF=\sum_{i=1}^{m}\left(Cost(i)+Mig(i)\right)$$

$$\;s.t.\left\{\begin{array}{c} \sum_{i=1}^{m}\delta_{i}^{uj}\leq R_{u}^{j}\\ \sum_{k=1}^{\kappa_{u}}c_{u}^{jk}\leq \phi_{i}^{uj}\\ \Phi_{1},\Phi_{2},\cdots ,\Phi_{m}\in \Phi \\ i=1,2,\cdots ,m\\ u=1,2,\cdots ,n \end{array}\right.$$

Specific to this optimized model, the article builds a synergistically distributed access model and further designs a Group Migration Bee Colony Optimized Scheduling algorithm (GMBCOS) to seek an optimal access strategy. Table 1 lists the symbols used in the model.

## 4. The Proposed Massive Sensor Network Data Access and Control Mechanism (MSACM)

When facing a massive number of access service requests, a traditional access means cannot easily meet the demand of low delay and high concurrence. As a result, a new strategy is needed to meet the constraint of users’ service quality and enhance the throughput and resource utility of the data center. Hence, based on the synergistically distributed buffering access model proposed, this section designs a Massive Sensor Network Data Access and Control Mechanism (MSACM), which first introduces an Optimized Dynamic Self-organizing Feature Map (ODSOFM) that divides the requests into groups dynamically; further, it provides a Group Migration Bee Colony Optimization Scheduling algorithm (GMBCOS) after analyzing the characteristics of the model of the existing virtual data center’s massive number of user’ requests; and finally expounds the steps of GMBCOS in detail and analyzes the performance of MSACM.

### 4.1. Optimized Dynamic Self-Organizing Feature Maps (ODSOFM)

#### 4.1.1. ODSOFM Introduction

**Figure 3:**
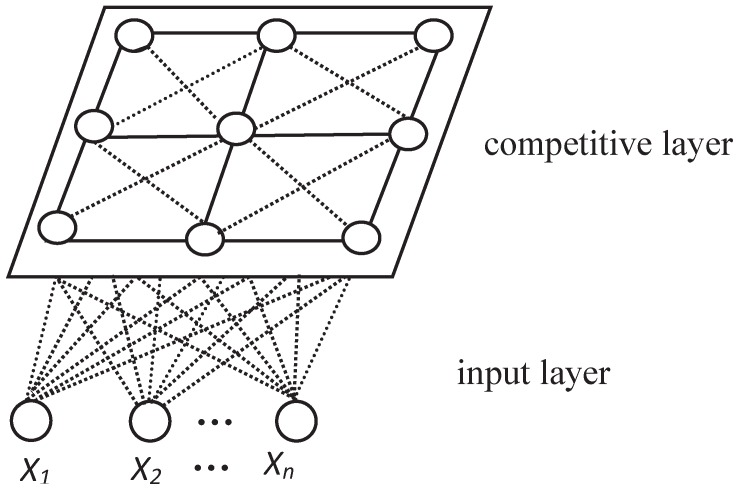
Topology of SOFM neural network.

A Self-Organizing Feature Map (SOFM) neural network, first initiated by Finnish scholar Kohonen, has been widely applied in a number of information processing fields [44] for its features, including topology structure maintenance, mass parallel processing, distributed information storage, good self-organizing and learning capacity, distributed adaptive output consistency [45], non-supervision of clustering and visualization, etc. SOFM is comprised of an input layer and output layer (the output layer is also called the competitive layer). The neurons of the input layer are fully linked to the competitive layer and each neuron represents one category. In accordance with the rules of learning, the repeated learning will be able to capture the feature of each of the input models, which will undergo self-organizing clustering and form a clustering result at the competitive layer. The topological graph is shown in Figure 3.

As shown in Figure 3, the input layer is made up of *N* input neurons while the competitive layer is made up of *M* output neurons. $X_{1},X_{2},\cdots ,X_{n}$ in the input layer refers to the *N* input neurons, and they are mapped into the output layer by clustering analysis. Neurons in the input layer and competitive layer are fully connected in the form of dotted lines. A precise grouping result can only be obtained by repeated self-organizing clustering for nodes in the competitive layer. When using a SOFM model, the number of neurons *M* at the competitive layer needs to be pre-designated. In this way, such a network structure will limit the network convergence speed to a large extent. This will be an obvious limitation. Therefore, in the scenario of the IoT’s mass access requests, in order to enable the users’ access requests to carry out self-organizing clustering based on their measurement nature, it is necessary to improve the SOFM, i.e., this requires an optimized ODSAOFM, which introduces a growing threshold (GT) and disturbance factor (DF). GT makes the network structure grow dynamically in the training process while DF controls the growth direction and prevents partial optimization, so that a hierarchical clustering is realized.

The algorithm of ODSOFM is as follows:

(5) $$GT=\left\{\begin{array}{c} \frac{d\times f(DF)\times n(lt)}{1+n(lt)}\;init=0\\ d\times f(DF)\;otherwise \end{array}\right.$$

where *d* is the dimension vector; init=0 refers to the initial status; n(lt) is the numbers of current network node at the lt-th time of iteration; f(DF) is the classification function of layers.

(6) $$f\left(DF\right)=\sqrt{1-DF}$$

DF=rand(lt)

where rand(lt) is the random number ranging from 0 to 1 at the lt-th time of iteration.

**Definition** **1.** *For vec, the network’s input dimension vector, the closest node on the competitive layer is regarded as the best matching node, and is abbreviated as bmn (best matching node). Hence, the formula is as follows:*

(7) $$∥vec-\omega_{bmn}∥\leq ∥vec-\omega_{n_{i}}∥,\forall n_{i}\in N$$

In the above equation, ω is the node weight dimension vector, $n_{i}$ is the ith network node, N is the total of $n_{i}$, ∥·∥ is Euclidean distance. $∥vec-\omega_{bmn}∥$ refers to the Euclidean distance between vector vec and vector $\omega_{bmn}$, which is the weight dimension vector of the best matching node bmn.

**Definition** **2.** *The distance between the input dimension vector vec and its best matching node bmn is referred to as the standard error value between them and is represented by E. Hence, the formula is as follows:*

(8) $$E=\sum_{k=1}^{d}{\left(vec_{k}-\omega_{bmn\;k}\right)}^{2}$$

In the above equation, d is the dimension of vec, $\omega_{bmn\;k}$ is the k-th dimension of the vector $\omega_{bmn}$.

**Definition** **3.** The competitive layer node $n_{i}$ and its direct sub-node are defined as the neighborhood of $n_{i}$, which is referred to as $\sigma (n_{i})$.

#### 4.1.2. Description of Optimized Dynamic SOFM

**Figure 4:**
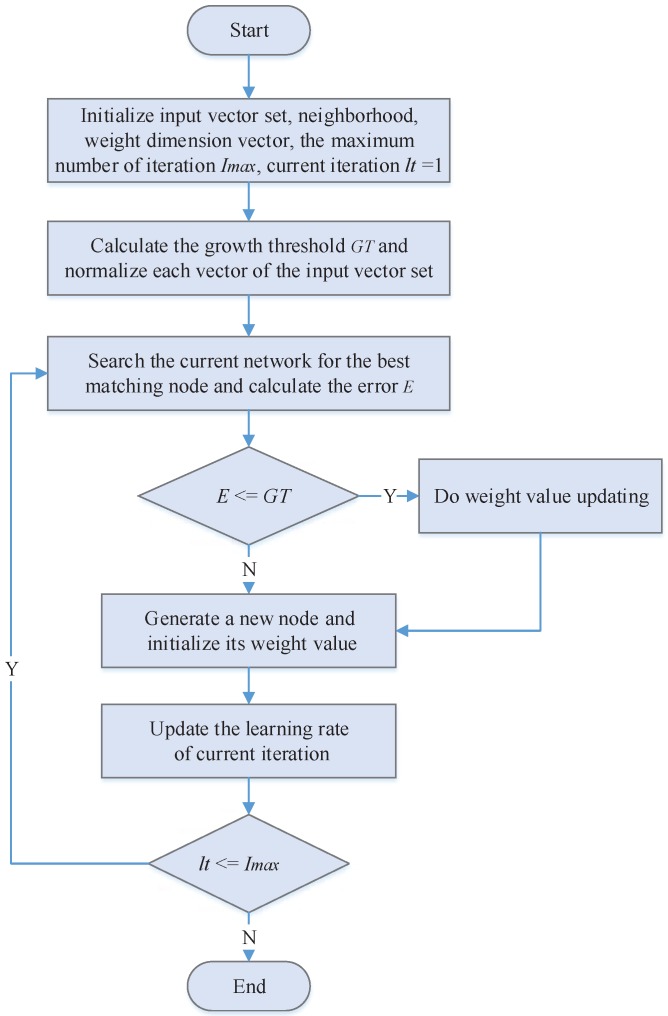
Flowchart of ODSOFM algorithm.

Given that $N=\{n_{0},n_{1},n_{2},\ldots ,n_{m}\}$ represents the node set at the competitive layer, $\;Vec=\{vec_{1},vec_{2},\ldots ,vec_{m}\}$ represents the input dimension vector set; $v_{i}=(vec_{i1},vec_{i2},\ldots ,vec_{id})$ represents *i*-th input dimension vector; *d* is the dimension of the input dimension vector; $n_{0}$ is the initial node; $\sigma_{k}\left(n_{i}\right)$ is the neighborhoods of node $n_{i}$ at *k*-th time of iteration, and $\sigma_{k}\left(n_{i}\right)=\sigma_{0}\exp\left(-\frac{k}{\tau }\right)$ ,$\sigma_{0}$ is the initial value of $\sigma_{n_{i}}$ (usually a bigger value), *τ* is exponential decay constant. This algorithm is summarized in the flowchart of Figure 4. Then, the detailed process of ODSOFM algorithm is as follows:

Step 1: Initiate node $n_{0}$, neighborhoods $\sigma (n_{0})$, weight dimension vector $\omega_{n_{0}}$, the maximum number of iteration $I_{max}$, and growth threshold GT, $\omega_{n_{0}}$ is a random value ranging from 0 to 1, at the initial period, lt=1, GT is calculated according to Equations (5) and (6), and then each vector of the input vector set Vec is standardized between 0 and 1. When it is $\bar{vec_{i}}=\left(\sum_{j=1}^{d}vec_{ij}^{2}\right){}^{\frac{1}{2}}$ then the standardized $vec_{i}$ is $\dot{vec_{i}}=\left(\frac{vec_{i1}}{\bar{vec_{i}}},\frac{vec_{i2}}{\bar{vec_{i}}},\ldots ,\frac{vec_{id}}{\bar{vec_{i}}}\right)$.
Step 2: Select the input dimension vector sequentially from Vec and search the current network node collection *N* for the best matching node (bmn) of the vector v, calculated by Equation (7);
Step 3: Calculate the error between bmn and *v* as *E* according to Equation (8). If E≤GT, skip to Step 4 for weight value updating, if not, go to Step 5 for the node growth operation;
Step 4: Adjust bmn neighborhood’s weight value via Equation (9).
  (9) $$\omega_{n_{j}}\left(k+1\right)=\left\{\begin{array}{c} \omega_{n_{j}}\left(k\right)\;j\notin \sigma_{k+1}\left(bmn\right)\\ \omega_{n_{j}}\left(k\right)+LR\left(k\right)\cdot \left(\dot{v}-\omega_{n_{j}}\left(k\right)\right)\;j\in \sigma_{k+1}\left(bmn\right) \end{array}\right.$$
  In the above equation, LR(k) is the learning rate, when k→∞, LR(k)→0 and $\omega_{n_{j}}\left(k\right),\omega_{n_{j}}\left(k+1\right)$ are the weight values of $n_{j}$ respectively prior and post adjustment. $\sigma_{k+1}\left(bmn\right)$ is the neighborhood when bmn is at k+1 times of iteration.
Step 5: Generate a new node $n_{p}$ of bmn, and make $\omega_{n_{p}}=\dot{vec}$;
Step 6: LR(t+1)=LR(t)×α, *α* is the regulating factor of LR, 0<α<1;
Step 7: Repeat Step 2 to Step 6 till all input dimension vectors in *V* have been trained completely;
Step 8: Make lt=lt+1, repeat Step 2 to Step 7 and enter into the next iteration period till no more new nodes are generated in the network.

### 4.2. Group Migration Bee Colony Optimization Scheduling Algorithm (GMBCOS)

The users’ requests are mapped into corresponding logical groups by using ODSOFM based on the distributed buffering access model, the analysis showing that the distributed solution of the ABC (Artificial Bee Colony) algorithm [46,47] features high efficiency, outstanding synergicity and is robust. A kind of group request migration scheduling algorithm is proposed based on the optimized ABC algorithm to search for the globally optimal solution.

#### 4.2.1. Basic Principle of the Artificial Bee Colony Algorithm

The ABC algorithm is a new intelligent algorithm based on the feeding behavior of bee colonies. Honey gathering is a process of searching for the optimal solution. A bee colony comprises three groups of bees: employed bees, onlooker bees and scout bees. Searching for the optimal solution involves basic behaviors such as searching for a honey source by the honey gathering bees and onlooker bees, calling for onlooker bees, and giving up the old honey source for a new one. In the process, the honey content of the honey source responds to the adaptability function of the optimization problem.

It is assumed that the initial group contains *N* solutions (the number of employed bees), each of the solutions $x_{i}$ is a dimensional vector, meaning the Number *i* location of a honey source; j∈{1,2,…,D} indicates the *j*-dimension component of solution vector. The detailed process of bee colony searching for the optimal solution is described as follows:

(1) Searching for honey source, in which the employed bee and onlooker bee search for a honey source in the manner shown in Equation (10):
  (10) $$v_{ij}=x_{ij}+\zeta_{ij}\left(x_{ij}-x_{kj}\right)$$
  In the equation, $v_{ij}$ refers to the new honey source location searched by Number *i* bee responding to Number *j* dimension, while $x_{ij}$ refers to the honey source location searched by current Number *i* bee responding to Number *j* dimension. $x_{ij}$ refers to Number *j* location of the randomly selected honey source *k*, k∈{1,2,…,D}, and k≠i. $\zeta_{ij}$ is the disturbance factor with a value being random between −1 and 1, which determines the search range for $x_{ij}$.
(2) Onlooker bee selects honey source. If the honey content of the new source is no less than that of the old one, then the employed bee accepts the location of the new source location, otherwise, it will continue to explore the old source. This can be understood as the employed bee determining a honey source by applying a greedy selection. When all the employed bees finish searching, they convey the information on the honey source to the onlooker bees, which will select the honey source based on the honey content and by means of Round Robin (RR). The equation for the selection probability is as follows:
  (11) $$p_{i}=\frac{F_{i}}{\sum_{n=1}^{N}F_{n}}$$
  In the equation, $P_{i}$ refers to the selection probability for Number *i* solution, and $F_{i}$ is the fitness value for Number *i* solution. This value corresponds to the honey mass at this point.
(3) Given the honey source and new solution is generated randomly, in case a specific solution is still not improved after $iter_{lim}$ times of recycling the iteration threshold, and the benefit degree of the honey source is not the global optimal solution, this indicates that the solution falls into a locally optimal solution and the solution should be given up. The employed bees responding to the solution should be changed to scout bees, which work to generate a new solution randomly to replace the old one as Equation (12) shows.
  (12) $$x_{ij}=x_{ij}^{min}+rand\left(0,1\right)\cdot \left(x_{ij}^{max}-x_{kj}^{min}\right)$$
  In the equation, rand(0,1) refers to the random numbers generated between 0 and 1, while $x_{ij}^{min}$ and $x_{ij}^{max}$ respectively refer to the upper and lower limits of Number *i* honey source of *j*-dimension component.

#### 4.2.2. Group Migration Bee Colony Optimization Scheduling Algorithm (GMBCOS)

It is understandable from the above paragraphs that the basic ABC represents such merits as fewer parameters for control, and easier computing and realization. However, it is easy for this to appear to be a “premature“ phenomenon, and the stride length of the search is largely random, causing the local search of the algorithm to be weaker and the convergence speed to be slower.

In this regard, the article introduces chaos theory and the particle location updating mechanism of the Particle Swarm Algorithm respectively with the purpose of improving ABC and thus solves the problem of DAPVDC when the migration of massive user’ requests occurs. This process is described in detail as follows.

(1) Chaos features randomness, ergodic property and regularity, enabling ergodicity of all kinds of status unrepeatedly within a specific rand and based on its own rule. With these characteristics, it is easy for chaos optimization to appear to be a locally optimal solution; and, if a specific solution is still not improved after $iter_{lim}$ times of recycling in ABC, this indicates that the solution falls into a locally optimal solution. In such a case, the scout bees will not select a new solution anymore according to Equation (12), but will generate a chaos sequence by utilizing the ergodic property of the chaos movement and based on the solution of the currently stagnant search. The optimal location in such a chaos sequence is going to replace the original location, causing the solution of the stagnant search to continue evolving, thus enhancing convergence speed and accuracy. The detailed steps are as follows:
  (a) To initialize a typical chaos system:
    (13) $$z_{n+1}=\mu \cdot z_{n}+\left(1-z_{n}\right)\;n=1,2,\ldots ,r$$
    where *r* refers to the sequential length, and *μ* refers to the control parameter. When μ=4, the system is in the status of chaos. By the time the value of *μ* is determined, an explicit chaos sequence $z_{1},z_{2},\cdots ,z_{r}$ can be iterated from an arbitrary initial value $z_{1}\in \left[0,1\right]$.
  (b) To assume that the solution for the current search stagnancy is $x_{i}$, which is mapped into the domain of definition [0,1] of the chaos system mentioned above as in the following equation:
    (14) $$z_{ij}^{1}=\frac{x_{ij}-x_{ij}^{min}}{x_{ij}^{max}-x_{ij}^{min}}\;i=1,2,\ldots ,N;j=1,2,\ldots ,D$$
    An iteration based on Equation (13) results chaos variable sequence $z_{i}^{\Phi }$ ($\Phi =1,2,\ldots ,iter_{lim}$), in which $iter_{max}$ refers to the maximum number of iteration for bee colony local search.
  (c) To bring chaos sequence $z_{i}^{\Phi }$ back to the original solution space via inverse mapping $x_{ij}=x_{ij}^{min}+z_{ij}^{m}\cdot \left(x_{ij}^{max}-x_{kj}^{min}\right)$, resulting in $x_{i}^{\prime }=\left(x_{i1}^{\prime },x_{i2}^{\prime },\ldots ,x_{iD}^{\prime }\right)$, and to calculate the adaptive value $F(x_{i}^{\prime })$ and compare it to the original, and maintain the optimal solution.
  (d) To complete the process of optimization if the local maximum number of iteration $iter_{lim}$, otherwise, return to Step b.
(2) In the process of searching for honey sources, employed bees and onlooker bees are merely searching through single-dimensional variables, and ignore the impact from variables of other dimensions, causing the convergence speed of the algorithm to reduce. Comparatively, in a particle algorithm, particles are update based on a globally optimal solution so that a better convergence speed can be maintained.

Inspired by this process, this article introduces a historically optimal location into the process of honey source searching. The new equation for the updated location is:

(15) $$v_{ij}=\left\{\begin{array}{c} x_{ij}+\zeta_{ij}\cdot \frac{F_{i}-F_{best}}{F_{k}-F_{best}}\cdot \left(x_{ij}-x_{kj}\right)+\lambda e^{-\frac{iter}{iter_{max}}}\cdot \left(x_{bestj}-x_{ij}\right)\;F_{k}\neq F_{best}\\ x_{ij}+\zeta_{ij}\cdot \left(x_{ij}-x_{kj}\right)+\lambda e^{-\frac{iter}{iter_{max}}}\cdot \left(x_{bestj}-x_{ij}\right)\;F_{k}=F_{best} \end{array}\right.$$

where $F_{best}$ refers to the adaptive value of the historically optimal honey source; iter refers to the current number of iteration; $iter_{max}$ refers to the maximum number of iteration; and *λ*, a parameter, is set as a constant. $x_{bestj}$ refers to *j*-dimension component for the current optimal solution.

The new location updating equation enables the globally optimal solution to play a guiding role to some extent, in the honey source search trend. What is more, it keeps a larger stride length at the initial period of iteration, which has benefit in extending the searching space of the algorithm, weakening the guiding role of the globally optimal solution and enhancing the algorithm’s convergence speed of the globally optimal solution.

**Figure 5:**
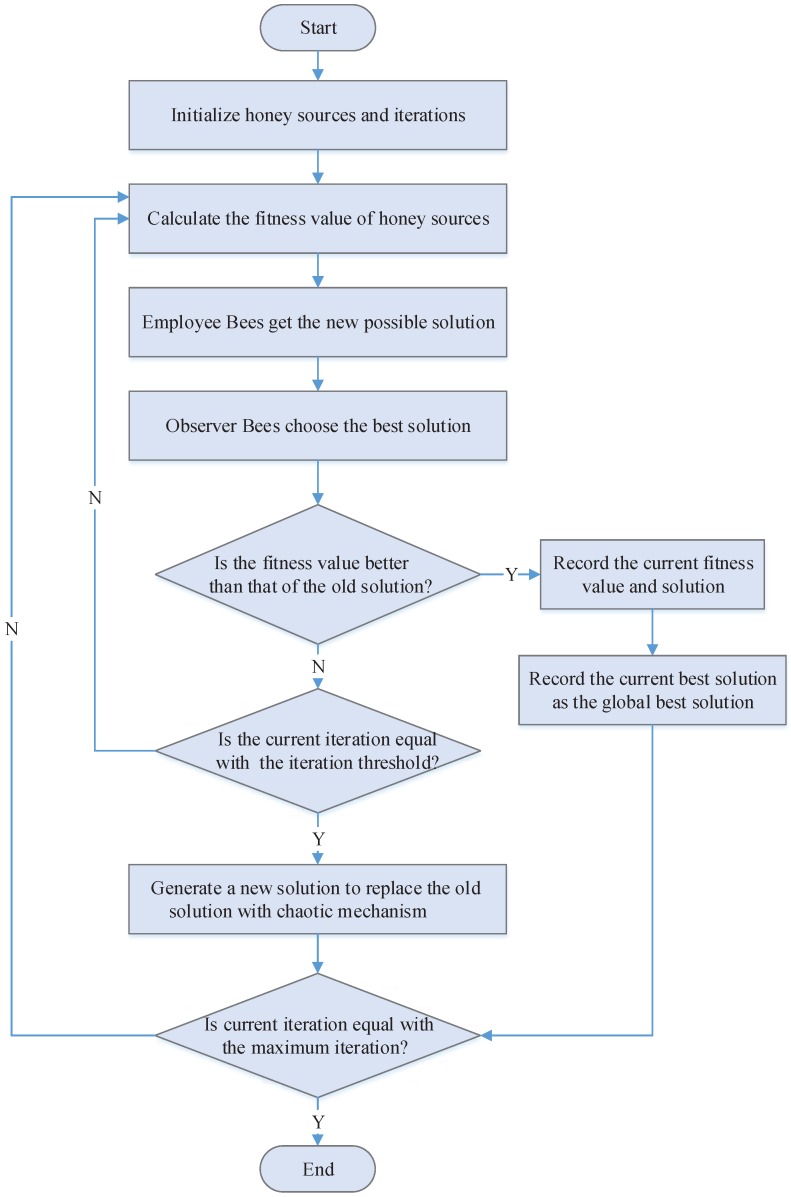
Flowchart of GMBCOS algorithm.

Using the analysis above, this article designs a Group Migration Bee Colony Optimization Scheduling algorithm (GMBCOS) based on the optimized ABC algorithm. This algorithm is summarized in the flowchart of Figure 5. In detail, the algorithm has the following steps.

Step 1: Initiation, to carry out logical grouping and efficiency verification of the users’ requests from different geographical locations;
Step 2: Randomly generate a number of initiative solution collection $x_{i}$, with the problem dimension as *D*, $iter_{lim}$ as the iteration threshold, *N* as the total number of honey sources, $iter_{max}$ as the maximum number of iteration, and iter=1;
Step 3: Calculate the fitness value of honey sources, $F(x_{i})$, and record the current optimal fitness value as $F_{best}$;
Step 4: Generate new locations according to Equation (15), and the employed bees select honey sources with the greedy selection mechanism;
Step 5: Onlooker bees select honey sources according to Equation (11);
Step 6: Onlooker bees generate new locations according to Equation (15) and select honey sources with the greedy selection mechanism;
Step 7: Give up the solution when it is still not improved (falls into locally optimal) after $iter_{lim}$ times of recycles continuously, and convert the employed bees corresponding to the solution to scout bees, which will generate a new solution to replace the old one according to the given chaos search mechanism;
Step 8: Record the optimal solution so far;
Step 9: iter=iter+1; judge if the condition for termination is met-if yes, terminate and output the optimal solution; and if not, go back to Step 3 for re-search.

### 4.3. Steps to Realize MSACM

Step 1: Initialize the DAPVDC problem, relevant functions and parameters, set the value of the relevant measurement parameters and constraint conditions for the DAPVDC problem, and move onto Step 2;
Step 2: Initialize the synergistically distributed buffer access model according to the parameters relevant to the DAPVDC problem, and move onto Step 3;
Step 3: According to the synergistically distributed buffer access model, use the ODSOFM algorithm to divide the massive access requests into dynamic groups based on the location characteristics;
Step 4: Use the GMBCOS algorithm to compute the model result regarding virtual logical requests, and move onto Step 5;
Step 5: Save the computed result and exit.

### 4.4. Analysis of MSACM Performance

The access scheduling strategy comprises two parts: the ODSOFM algorithm and GMBCOS algorithm. For the former, the growth threshold (GT) determines the network growth scale. When GT is comparatively large, ODSFM will have proportionally more weight value updating operations and generates less joints to be included in the target network, yet the speed of generating the network is faster and only realizes the clustering of coarse particles. In contrast, when GT is comparatively small, ODSFM will have proportionally more joint growth operations and generates more joints to be included in the target network, yet the speed of generating the network is slower and only realizes the clustering of fine particles. When the disturbance factor (DF) is introduced, it is always set to a larger value for initiation. It can be known from Equation (6) that GT at this moment is smaller and is able to categorize the transmission orders roughly, enabling an overall grasp of the group transmission order. With an increasing number of iterations, the value of DF grows larger, enabling more accurate results on clustering and realizing clustering in layers. Additionally, it is known from Equation (5) that the increasing number of iterations results in to GT having a weaker role in adjustment, more weight value updating operations, being able to more easily select the properties that have a major function in the clustering result and ignore the properties with minor functions. Furthermore, this will reduce the computational work and further enhance the algorithm’s convergence speed and execution efficiency.

The optimized ABC-based algorithm, GMBCOS, introduces the fitness value that is so far the optimal honey source to enhance the global development capacity, and the search radius appears to be a ladder-type variation for developing the optimal solution as the number of iteration grows. This balances the exploration for the optimal capacity and development capacity well, and the algorithm presents excellent convergence and viability. This algorithm shows an identifiable role division among the three kinds of bees—the employed bees work to maintain the optimal solution; the onlooker bees enhance convergence speed; and the scout bees enhance the capacity to get rid of the locally optimal solution. When the scout bees are updating the stagnant solution, the article algorithm uses a chaos search mechanism to increase the solution diversities, continuing to optimize the stagnant solution and further enhancing the convergence speed and accuracy.

Regarding the complexity of executing the MSACM mechanism, the number of iterations is $I_{max}$; the number of input nodes is *N*; the maximum number of iteration operations of each round is NlogN; the maximum space occupation is *N*; the overall time complexity after $I_{max}$ times of iteration is $o(I_{max}\times NlogN)$; and the space complexity is o(N). However, the number of initial solutions for GMBCOS is *N*. A chaos sequence with a length of *r* will be generated via chaos search every time when the chaos search is being performed, with r<<N. The maximum execution number of each iteration of the employed bees and onlooker bees is NlogN; the maximum globally executing number is $iter_{max}$, the total number of search executed by the algorithm is $NlogN\times iter_{max}$. Hence, the overall space complexity of the MSACM mechanism is o(N) and the time complexity is $o(Max\left\{I_{max}\times NlogN,NlogN\times iter_{max}\right\})$. In the real process of operation, as $iter_{max}$ is bigger than $I_{max}$, so the time complexity of MSACM is $o(NlogN\times iter_{max})$ and the space complexity is o(N).

## 5. Performance Evaluation

### 5.1. Experiment Environment and Parameter Setting

The experiment platform is mainly composed of three groups of low-cost clusters, namely, sending server cluster, access server cluster and storing server cluster. The sending server cluster primarily works to simulate sensor nodes to send massive sensor data, with each sending server deploying the same program at the sending end and controlling sensor data of various size and rate. The sensor data all bear area characteristics, for which the sending server cluster carries out parallel control via the MPI (Message Passing Interface) environment. The access server cluster works on access processing for the massive sensor data. The access cluster is regarded as a virtual data center. Each of the servers deploys the MSACM mechanism, and Hadoop and Ganglia software. The entire access cluster manages the resources via Hadoop and monitors the resource variation via Ganglia. The storing server cluster stores the data processed by the access server. Each server deploys HBase software. This a kind of distributed storage of the massive structured or non-structured sensor data processed by the access server.

**Figure 6:**
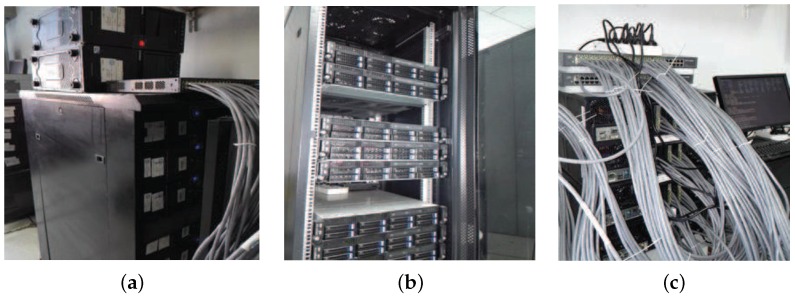
Grouping of server cluster. (**a**) Sending server cluster; (**b**) Storing server cluster; (**c**) Access server cluster.

The sending server cluster consists of 8 physical machines, each configured with a 4-core CPU, Intel(R) Core(TM) i5-3470 CPU @ 3.20 GHz, 4 G memory, 250 G hard disk, 1000 M network card, 64-bit CentOS-6.4. The deployment structure is shown in Figure 6a. The storing server cluster consists of 8 physical machines, each configured with 8-core CPU, Intel(R) Xeon(R) E5-2603 v2 @ 1.80 GHz, 16 G memory, 2 T hard disk, rotating speed 7200 rpm, 1000 M network card, 64-bit CentOS-6.4. It is configured with an HBase distributed storing database, each of the storing nodes being configured with Hadoop-2.6.0, HBase-0.96 and zookeeper-3.4, and also Ganglia-3.7.1 software to detect the resource utilization situation at the nodes. The deployment structure is shown in Figure 6b. The access server cluster consists of 16 physical machines connected with a network cable of 7 categories and 2 sets of 1 GB D-Link Ethernet Switch, each configured with 8-core CPU, Intel(R) Core(TM) i7-4790 CPU @ 3.60 GHz, 40 G memory, 12 pieces of 1000 M network card, and 1 T hard disk. The deployment structure is shown in Figure 6c. These 16 physical machines are arranged with 64 virtual servers to compose a virtual data center. Each of the virtual machines is configured with 8 G memory, 4-core CPU, 200 G hard disk, 64-bit CentOS-6.4, and Hadoop-2.6.0, Ganglia-3.7.1 for resource management and monitoring. These virtual machines are divided into a few groups based on their location property. The experiment builds up a sending server cluster with 8 groups of physical servers, each group with 2 sets of Linux virtual machines. These 16 sets of Linux virtual machines simulate the sending request user end from different areas. Each of the user ends sends a specific amount of access requests via the multi-threading sending program. These massive access requests from various areas send service requests regularly toward the access cluster servers at the virtual data center. A single distributed buffering access system is arranged to a virtual server selected from each of the access server groups to inquire the mapping relation between the logical request group and the virtual server. The MSACM mechanism is deployed for each virtual access server. In the end, a distance control system is applied to control the 16 local client hosts sending their service requests of different mass and thus build up a massive access environment for the IoT. Each virtual client host opens a number of service request threading based on the test scale. Each thread sends a number of service request packets, each with a size of 128 bytes.

With the comprehensive consideration of the real scenario of massive request access, it is initialized that the number of solution N=30, and the request order dimension ranges from 3 to 6. A larger request order dimension will require higher difficulty in computing and it will be easier to test the bottleneck of the algorithm. In this case, with the maximum dimension value being set at D=4, the growth threshold (GT) and disturbance factor (DF) can be calculated according to Equations (5) and (6). The initiative neighborhood radius reflects the request order’s range of clustering. At the initiative, the order categorization is always indistinct, as a larger value is set for $\sigma_{0}$, which, however, has little impact on the clustering result, as different initiative domain may acquire the same result even after different times of iteration, with $\sigma_{0}=12$. The adjustment factor *α* of the Learning Rate (LR) reflects the degree of ease of the request order clustering when iteration times of service request order increase. With 0<α<1, a larger *α* indicates that the request command more easily finds its category. It ranges from 0.6 to 0.9 according to the characteristics of the service requests’ scale of simulated users. Without loss of generality, in this paper, we set α=0.75 according to the literature [48], and set $iter_{max}=60$, $iter_{max}=200$ according to the literature [49].

### 5.2. MSACM Performance Test

The test commences with the experimental environment and parameters set as aforementioned. Without loss of generality, both algorithms are executed ten times independently to obtain the average statistical results in each experiment. The experiment is designed with consideration of the following aspects:

(1) In the situation of steadily increasing service request speed, the request arrival rate (RAR) is compared between MSACM and the other algorithms. RAR is defined as: RAR = number of service requests with successful arrival/total number of service requests;
(2) In the situation of steadily increasing service request speed, network delay (ND) is compared between MSACM and other algorithms. ND herein refers to the average network delay;
(3) In the situation of steadily increasing number of access service requests, the load balance rate (LBR) is compared between MSACM and other algorithms. The load of Number *i* access server at the moment of *t* is set as $\pi_{i}$, the total number of online virtual machines is *κ*, the average load of the access cluster is $\bar{\pi }=\frac{\pi_{1}+\pi_{2}+\cdots +\pi_{\kappa }}{\kappa }$, and the load balance rate is defined as $LBR=\frac{1}{\kappa }\sum_{i=1}^{\kappa }{\left(\pi_{i}-\bar{\pi }\right)}^{2}$;
(4) In the process of a gradually growing number of virtual servers, the resource utilization rate (RUR) is compared between MSACM and other algorithms. The number of online virtual machines is set as *κ*, the time consumed by Number *j* access servers when requesting access is $AT_{j}$, the real occupation time of Number *j* access servers is $OT_{j}$, then RUR is defined as $RUR=\frac{1}{\kappa }\sum_{j=1}^{\kappa }\frac{AT_{j}}{OT_{j}}$;
(5) In the situation of a steadily growing number of service requests, the migrating cost rate (MCR) is compared between MSACM and other algorithms. The number of online virtual machines is set as *κ*, the migrating time consumed by Number *j* access servers when requesting access is $MT_{j}$, and the time consumed by Number *j* access servers when requesting access is $AT_{j}$, then MCR of the access server cluster is defined as $MCR=\frac{1}{\kappa }\sum_{j=1}^{\kappa }\frac{MT_{j}}{AT_{j}}$.

#### 5.2.1. Experiment 1: RAR Performance with Different Request Rate

In this experiment, RAR performance is tested under the condition that the maximum request rate is 1 million packets per second and the minimum is 0.82 million packets per second. In order to evaluate RAR performance, the experiment uses the WRR [43] algorithm, which is based on Round-Robin Scheduling, Kafka [50], which is a distributed message publishing and subscribing mechanism, and the MSACM algorithm proposed in this paper.

**Figure 7:**
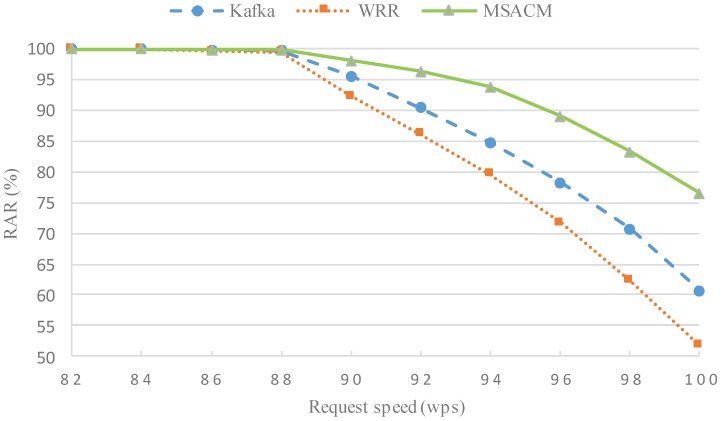
RAR with different request speed.

In Figure 7, the horizontal coordinates refer to access service request speed; wps refers to ten thousand access request packets sent by clients per second; and the vertical coordinates refer to the request arrival rate. When the request speed remains below 88 wps, the network resources and computing resources in the virtual data center are plentiful, WRR and Kafka show a satisfactory access performance, without the occurrence of packet loss. When the request rate is 90 wps, the RAR of WRR algorithm is 92.38%, the RAR of Kafka is 95.67% and the RAR of the MSACM algorithm is 98.12%. However, with a rising request speed, the network bears a larger load and network links start being blocked and packet loss begins at the virtual data center. This phenomenon is especially apparent in WRR and Kafka. When the request rate reaches 94 wps, MSACM still maintains a request arrival rate of 93.85%, whereas the other two algorithms have a request arrival rate lower than 85%. With ever growing request concurrency, virtual servers have a high consumption of resources, with weakening communication capacity between virtual machines and an unapparent scheduling effect of both algorithms. Gradually, the request arrival rate of the three algorithms becomes largely different—MSACM performs well while the Round-Robin Scheduling-based WRR algorithm has the lowest rate. In the process of the request speed gradually increasing from 82 wps to 100 wps, the RAR of the MSACM algorithm changes quite gentle, which shows that MSACM algorithm has a good robustness. This also shows that Kafka, an outstanding distributed message system, cannot effectively solve the distributed access problem for virtual data centers at the DACIOT.

#### 5.2.2. Experiment 2: ND Performance with Different Request Rate

In this experiment, the network delay (ND) variation of the three algorithms is tested when the access request speed varies from 0.82 million to 1 million packets per second.

**Figure 8:**
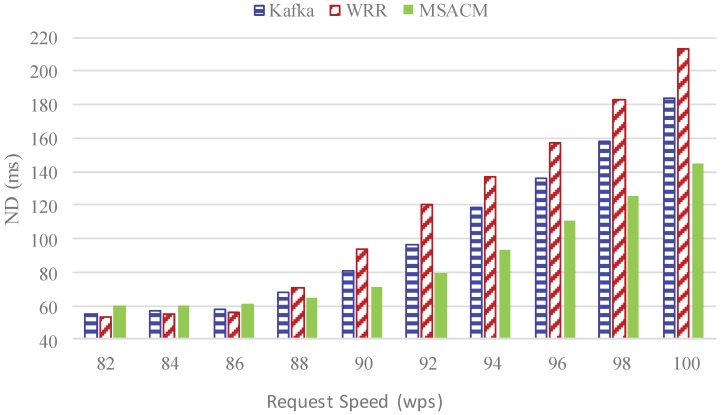
Network delay of various request rate.

In Figure 8, the horizontal coordinate refers to the access service request rate while the vertical coordinate refers to average network delay. In the process of the request speed gradually increasing from 82 wps to 86 wps, it can be seen from the figure that the load at each access server remains low and network link congestion does not appear as the access cluster enjoys ample network resources when the access service request rate is low. Network delay mainly refers to the transmission time. In this sense, the network delay of the three algorithms is all low and at similar levels. Strictly, WRR is more slightly advanced than the other two algorithms. As MSACM needs to use ODSOFM algorithm to classify the access requests, it shows a slightly longer delay as there is more frequent transmission of virtual machines. Nevertheless, with an ever growing request rate, the access server consumes resources at a faster pace; network bandwidth has less resources; service requests require a longer time; and thus the average network delay increases. When the access service request rate surpasses 86 wps, the three algorithms clearly start to differ from each other; and when it reaches 88 wps, Kafka has a shorter network delay than WRR. When the request speed increases to 94 wps, the average network delay of MSACM is 93 ms, and the average network delay of WRR reaches 137 ms, and the average network delay of Kafka reaches 118 ms. With the ever growing pressure from the access servers, the time to process the service request and network transmission gradually starts to increase, and the three algorithms start to differ from each other clearly in the network delay. Generally, however, MSACM has a shorter average network delay than the other two algorithms. It also has a slower variation, indicating better stability.

#### 5.2.3. Experiment 3: LBR Performance with Variable Request Scale

In this experiment, the variation of the load balance rate (LBR) of the access server cluster of the three algorithms is tested when the service request number grows gradually from 50 million packets to 100 million with a stride length of 5 million.

**Figure 9:**
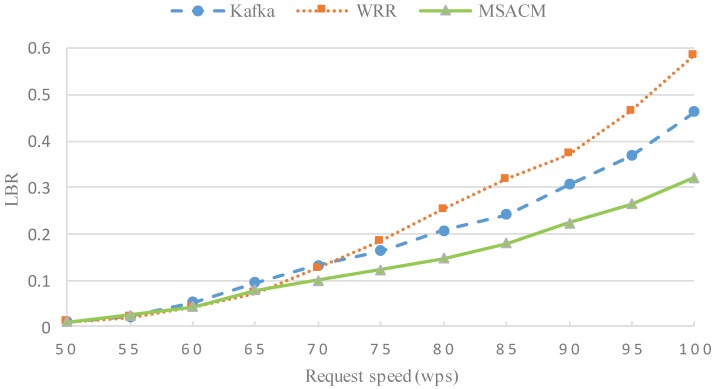
LBR of various number of access service request.

In this experiment, as shown in Figure 9, the horizontal coordinates refer to the number of service requests with a unit of 1 million packets; and the vertical coordinates refer to the load fluctuation rate. When the service request number is below 60 million, the virtual access cluster server can deal efficiently with the service requests and the virtual data center does not have pressure from network communication and resource consumption. In the experiment, the three algorithms all have a lower LBR. In addition, as MSACM has consumed some network bandwidth resources when it continues inquiring the distributed buffering system at the initial period, LBR is slightly higher than the Round-Robin Scheduling-based WRR. When the number of service request increases to 65 million, the LBR of MSACM algorithm has a better performance than Kafka and WRR, which reflects the advantage that MSACM utilizes ODSOFM algorithm to group the service requests. With the ever growing number of service requests, the data center experiences larger pressure and frequent scheduling. In this experiment, the three algorithms present increasing LBR and jitter on different levels. When the request number reaches 75 million, WRR has the highest LBR, followed by Kafka and MSACM. Later, as WRR requires frequent task scheduling, communication between the virtual machines grows, so WWR shows the lowest LBR. On the other side, Kafka shows a sharply weakening LBR along with an increasing number of service requests. As network communication reduces in the buffering mechanism, MSACM has better LBR than both Kafka and WRR.

#### 5.2.4. Experiment 4: RUR Performance with Variable Access Servers

In this experiment, variation of resource utility rate (RUR) of the data center is tested when the number of virtual access servers varies from 30 to 64 and the request rate remains at 88 wps.

**Figure 10:**
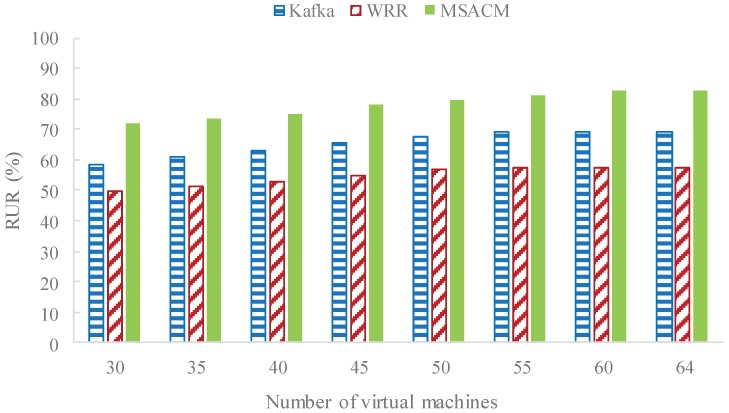
Resource utility rate of different numbers of online virtual machines.

In Figure 10, the horizontal coordinates refer to the number of switched-on virtual machines, and the vertical coordinate refers to data center’s RUR. It can be seen from these three experiments that the three algorithms all have high access request acceptability when the access request rate remains at 88 wps, which is when the performance of each differs. This is the reason why the experiment tests the impact of the number of online virtual machines on RUR under the condition of an appropriate access request rate. In the beginning, the effective resources of the virtual data center is the bottleneck of the current massive service requests, the algorithm of WRR, Kafka and MSACM are lack of computing resources, which lead to the service request coming about more resources competition. As the experimental results shows, when the number of access virtual machines is below 50, the amount of resource requirements is not satisfied. With an increasing number of access virtual machines, each computing tasks in the virtual data access center can be assigned more CPU and memory resources, and the time to complete the service access requests gradually reduces. In this experiment, the data center’s RUR of the three algorithms increases gradually, however, the scheduling strategy of MSACM has obvious advantages, and so the RUR of virtual data center is slightly higher in MSACM. With a further increase in the number of virtual machines, the impact on communication time gradually weakens; with adequate resources, the time to complete the service access requests is no longer increasing; and the time variation of access to the occupied virtual machines is also weakening. Therefore, the RUR of the three algorithms no longer varies. From the perspective of RUR, MSACM has better convergence than the other two algorithms.

#### 5.2.5. Experiment 5: MCR Performance with Variable Request Scale

In this experiment, the variation of the migration cost rate (MCR) of the access server cluster of MSACM is tested when the number of service requests grows gradually from 50 million packets to 100 million with a stride length of 5 million.

**Figure 11:**
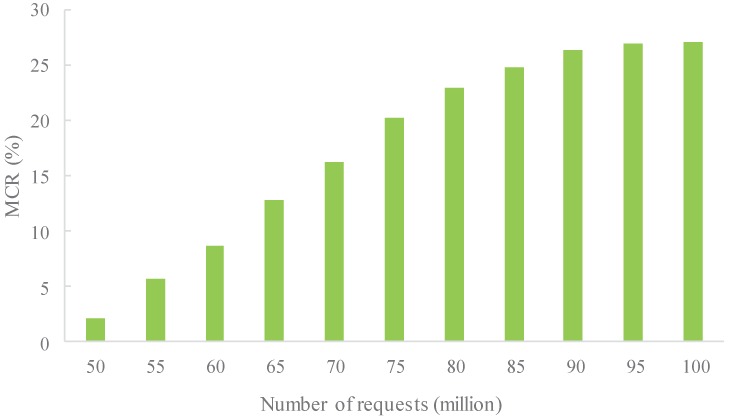
Migration cost of varied number of access service requests.

In Figure 11, the horizontal coordinates refer to the number of service requests with a unit of 1 million packets; and the vertical coordinates refer to migration cost. Migration consists of both data migration and status migration. It can be seen from the figure that the migration cost value is only 2.11%, implying a very short time occupied by migration. At this moment, very few access nodes have the request for migration as the processing capacity of the access nodes in the access cluster can accommodate the small number of access requests. The consumption cost mainly lies in access processing. At this stage, MSACM mechanism takes advantages of ODSOFM algorithm to group different types of service requests, then with the cache model, MSACM is able to quickly dispatch these grouped requests to the corresponding access server for processing. Furthermore, the whole process occupies less network resources and computing resources of the virtual data center. As the request number continues to grow, resource consumption including network bandwidth and CPU also grows gradually and part of the access nodes reach the threshold of their processing capacity. At this moment, the migration of data and status between nodes starts to grow. In the process of migration, the status migration consumes large amounts of memory resources, and the data migration consumes more bandwidth resources. In the case of massive service requests, MSACM needs to use the scheduling policies frequently for migration, which will slow the network congestion. When the request number reaches 75 million, MCR has already reached 20.16%. The time consumed by migration affects the time to complete the request access. However, the migration cost varies very little as the request number further grows. This indicates that the scheduling strategy of MSACM enables the access cluster to reach a reasonably good status of balance. Moreover, each of the access nodes is able to execute the access processing task in a balanced manner. This indicates that MSACM has a comparatively good coordinative migration capacity and is robust.

#### 5.2.6. Analysis

The above experiments evaluate the performance of MSACM from the aspects of request arrival rate, average network latency, load fluctuation rate, resource utilization and migration costs. Under the condition of the same request speed, experiment 1 and experiment 2 respectively test the request arrival rate and the average network delay of WRR, Kafka and MSACM. The above two experimental results in Figure 7 and Figure 8 show that, due to the load effect of the distributed cache access model, MSACM can guarantee all of the service requests access to the virtual data center by dynamically scheduling the resources of virtual data centers, and the network delay is less than 80 ms when there are sufficient network resources. In general, when the request speed remains below 90 wps, MSCAM can sustain the communication resource consumption of service requests, and keep more than 98% of the request arrival rate. From a more global perspective, MSCAM has a better performance than Kafka and WRR under the condition of existing resources in the virtual data center.

Combined with the analysis shown in Figure 9 and Figure 10, the communication between server nodes in the virtual data server varies with the change of the number of service requests, and the network resource in the virtual data center fluctuates as well. Moreover, the scheduling strategy of MSACM can promote the performance of the load conditions and resources optimization. In the case of high concurrency, MSACM’s swarm scheduling algorithm can effectively transfer the service requests from the service access node of the higher load pressure to that of the lower load pressure, thus maximizing the use of the virtual data center’s resources. In the aspect of load-balance evaluation, depending on the caching mechanism, MSACM can record the history optimal scheduling path, and reduce the network communication, which reflects the optimal load balancing ability. Furthermore, Figure 11 shows that MSACM can effectively control the migration cost. When the number of service access requests is growing, the proportion of the time consumed for task migration and the time consumed for service access gradually stabilizes, which shows the advantage of request grouping. This also shows that MSACM has good coordination capacity and robustness in respect to request migration. When there are very limited network resources and limited physical resources of the access cluster, the MSACM mechanism is more efficient than the other methods (WRR and Kafka) when solving the problem of large-scale service request distributed access to virtual data center.

## 6. Conclusions

The Data Access Center for the Internet of Things (DACIOT), as the end of the sensor network application, plays a determinative role in massive sensor data access and control. Modern new-type data centers are primarily supported by virtualization technology, which is an important means to enhance resource utility. In a virtual environment, focusing on the massive amount of access requests for sensor data in different locations, this paper first designs a distributed buffering access model with a separation between location and information. Then, this paper utilizes an improved self-organizing feature map neural network (SOFM) for dynamic grouping according to the location information. Further, CPU, memory and network resources stored in the virtual data center are unified in an abstractive manner. The paper also proposes a group migration bee colony optimization scheduling algorithm based on the artificial bee colony algorithm (ABC). In terms of solving the problem of the Internet of Things’s large-scale service request distributed access to virtual data center, this algorithm can enhance the request arrival rate (RAR) with shorter average network delay and better load balance capacity and resource utility. Moreover, MSACM has a good coordinative migration capacity and is robust in relation to request migration.

Further work will cover an extension of the proposed access model, on the basis of which, we will study in-depth the large-scale distributed service request queues of TCP and the UDP flow congestion control algorithm. Future work will also take into consideration the fairness of service request access.

## Figures and Tables

**Table 1:** Parameters of the DAPVDC modelling.

Parameters	Descriptions
*G*	Collection of virtual logic groups of user’s requests
*V*	Collection of virtual machine groups in data center
*P*	Collection of physical servers in data center
*L*	Collection of network links in data center
$p_{a}$	The number *a* of physical servers
S(a)	If the physical server $P_{a}$ works well
*s*	Number of physical servers
$g_{i}$	The number *i* logic groups of users’ requests
$\upsilon_{u}$	The number *u* groups of virtual machines
$\kappa_{u}$	Number of virtual machines in the group of virtual machines $\upsilon_{u}$
$\upsilon_{u}^{j}$	The number *j* virtual machines in the group of virtual machines $\upsilon_{u}$
$R_{u}^{j}$	Amount of resources needed by virtual machine $\upsilon_{u}^{j}$
$Q_{a}$	Gross resources of physical server $P_{a}$
$c_{u}^{jk}$	Communication resources between virtual machines $\upsilon_{u}^{j}$ and $\upsilon_{u}^{k}$ in the time of Δt
$\psi_{i}^{uj}$	Network communication resources consumed when request group $g_{i}$ is accessed by virtual machine $\upsilon_{u}^{j}$
$\tau_{u}^{j}\left(i\right)$	If request group $g_{i}$ is successfully accessed by virtual machine $\upsilon_{u}^{j}$
$\varphi_{j}^{k}\left(i\right)$	If request group $g_{i}$ migrates from virtual machine $\upsilon_{u}^{j}$ to $\upsilon_{u}^{k}$
S(i)	Measurement of size of request group $g_{i}$
*λ*	Adjusting factor that balances impact of other resources on request migration
$\Phi_{i}$	Viable access means of request group $g_{i}$
